# Vulnerability of Older Adults to Government Impersonation Scams

**DOI:** 10.1001/jamanetworkopen.2023.35319

**Published:** 2023-09-22

**Authors:** Lei Yu, Gary Mottola, Christine N. Kieffer, Robert Mascio, Olivia Valdes, David A. Bennett, Patricia A. Boyle

**Affiliations:** 1 Rush Alzheimer’s Disease Center, Rush University Medical Center, Chicago, Illinois; 2 Department of Neurological Sciences, Rush University Medical Center, Chicago, Illinois; 3 FINRA Investor Education Foundation, Washington, DC; 4 Department of Psychiatry and Behavioral Sciences, Rush University Medical Center, Chicago, Illinois

## Abstract

**Importance:**

Financial fraud and scams targeting older adults are on the rise and pose serious public health and economic threats. Research on the vulnerability of older adults to fraud and scams relies almost exclusively on self-reported data, which have several intrinsic limitations. Thus, how older adults truly respond to fraud attempts remains unclear.

**Objective:**

To explore the vulnerability of older adults to a US government impersonation scam.

**Design, Setting, and Participants:**

This cross-sectional study, conducted from October to December 2021 among communities in the greater Chicago metropolitan area, was framed as a fictitious government agency reaching out to older adults about a potential compromise of personal information relevant to their Social Security and Medicare benefits. Participants were older adults participating in the Rush Memory and Aging Project, an ongoing cohort study of chronic conditions of aging. Data analysis was performed from February to August 2023.

**Exposures:**

Participants were exposed to deceptive materials through mailers, emails, and phone calls by a live agent.

**Main Outcomes and Measures:**

Based on the phone call data, participants were classified into 3 groups: no engagement (participants who did not answer the phone or call in), engagement (those who answered or called in but were skeptical about the legitimacy of the outreach and did not give away personal information), and conversion (participants who answered or called in without skepticism, or confirmed that they did not change their personal information, or provided the last 4 digits of their Social Security number).

**Results:**

A total of 644 older adults (501 [77.8%] female, 143 [22.2%] male), with a mean (SD) age of 85.6 (7.5) years, were included. A total of 441 (68.5%) participants did not engage, 97 (15.1%) engaged but raised skepticism, and 106 (16.4%) converted. Older adults who engaged but with skepticism had the highest cognition and financial literacy, while those in the conversion group had the lowest scam awareness. No differences were observed in psychological and other behavioral measures by the levels of engagement.

**Conclusions and Relevance:**

In this cross-sectional study using a behavioral experiment that mimicked a real-world imposter scam, a sizable number of older adults engaged without skepticism. Results suggest that many older adults, including those without cognitive impairment, are vulnerable to fraud and scams.

## Key Points

**Question:**

How vulnerable are older adults to government impersonation scams?

**Findings:**

In this cross-sectional study using a behavioral experiment designed to mimic a real-world imposter scam among 644 older adults, a sizable number of older adults (16.4%) engaged without skepticism, and of those, nearly three-quarters provided potentially compromising personal information. Analyses revealed that cognition, financial literacy, and scam awareness were associated with vulnerability.

**Meaning:**

Many more older adults than currently recognized, including those without cognitive impairment, are vulnerable to fraud and scams, placing them at considerable risk for adverse health and financial outcomes.

## Teaser

This cross-sectional study explores the vulnerability of older adults to a US government impersonation scam.

## Introduction

Financial fraud and scams that target older adults present major public health and economic challenges. It is estimated that each year, 1 in 18 cognitively intact older adults in the US falls victim to financial fraud and scams,^1^ and the number is thought to be much higher when considering individuals with cognitive impairment in the general aging population. The dollar losses to financial fraud and scams are incredibly high. According to a report released by the US Senate Special Committee on Aging, in 2020, older Americans lost $100 million to COVID-19–related frauds alone. The latest sentinel data collected by the Federal Trade Commission found that older adults filed close to half a million fraud reports in 2022 with a collective loss of over $1.5 billion.^2^ A report by AARP paints a gloomier picture, estimating that each year, stranger-perpetrated fraud costs older adults over $8 billion.^3^ Fraud victimization also takes a toll on physical health and psychological well-being and is associated with hospitalization, loss of independence, depression, suicide, and early mortality.^4,5,6,7^ This is particularly concerning for older adults because they have limited capability to recover from these adverse consequences. The public health and economic challenges presented by fraud and scams will only intensify as our nation continues to age.

A key step toward protecting older adults from financial fraud and scams is to gain a better grasp of the scope of the problem, which remains elusive. To date, data on fraud victimization come almost exclusively from complaints filed with government agencies or surveys.^8^ Survey data are widely used by the research community and provide valuable information on the prevalence, determinants, and consequences of financial fraud and scams.^1,9,10,11^ However, because surveys rely on older adults’ ability to recognize, admit, and report fraud, survey data have intrinsic limitations that can range from recall bias to underreporting due to fear, shame, or lack of awareness that one has been victimized.

In collaboration with the Financial Industry Regulatory Authority (FINRA) Investor Education Foundation, we conducted a behavioral experiment designed to mimic a government impersonation scam. The experiment involved a fictitious government agency reaching out to community-living older adults about a potential compromise of personal information relevant to individuals’ Social Security and Medicare benefits. Participants were contacted through mailers, emails, and phone calls by a live agent. The current study focused on data from phone conversations with the live agent, as many participants did not have valid emails on file or internet access. Our primary aim was to assess the vulnerability of older adults to government impersonation scams.

## Methods

### Study Participants

Participants came from the Rush Memory and Aging Project (MAP), an ongoing cohort study of common chronic conditions of aging.^12^ Starting in 1997, investigators at the Rush Alzheimer’s Disease Center have worked with local communities, churches, and senior centers to recruit older laypersons throughout the greater Chicago metropolitan area. Most participants are residents of continuous-care retirement communities, subsidized housing, or retirement homes. Others enrolled through churches and social service agencies. Participants agreed to annual clinical evaluations, which include cognitive assessments. In 2010, a decision-making substudy was added to investigate financial and health decision-making behaviors in older age. The parent study, and separately the decision-making substudy, were each approved by an institutional review board of Rush University Medical Center. All participants provided written informed consent and a repository consent for data sharing.

The behavioral experiment was conducted from October 1 to December 31, 2021. By October 1, 2021, 1292 MAP participants had completed the baseline evaluation of the parent study as well as the decision-making assessment, 521 had died, 76 had withdrawn from the study, and another 24 had declined further contact. The materials were sent to 644 of the remaining 671 participants who were alive and active (eFigure 1 in Supplement 1). The experiment was reviewed and approved by an institutional review board of Rush University Medical Center. Due to its deceptive nature, we did not seek additional informed consent. Instead, the experiment was to be followed by a debrief that included a discussion with participants about the study, as well as a presentation by a FINRA Investor Education Foundation representative on financial fraud and scams. The debrief was disrupted due to the COVID-19 pandemic and is in process. Findings in the current study were reported following the Strengthening the Reporting of Observational Studies in Epidemiology (STROBE) reporting guideline for cross-sectional studies.

### Design of the Behavioral Experiment

Government impersonation scams were one of the top 5 scams reported to the US Senate Special Committee on Aging between 2015 and 2020. Therefore, we developed materials for a fictitious US Retirement Protection Task Force (USRPTF), purporting to be a government agency that handles important government files essential to Social Security and Medicare benefits. Participants were told that there was unusual activity on their file and that the agency was reaching out to verify that the activity was authorized. This formed the backdrop for asking participants for personal information and closely mimicked the tactics used by fraudsters.

The experiment comprised 3 outreach strategies including mails, emails, and phone calls by a live agent. Specifically, on October 6, 2021, and November 5, 2021, a piece of mail (eFigure 2 in Supplement 1) was sent to each participant’s address. The mailer claimed that the agency had made multiple attempts to contact the individual about a possible breach of their account and required them to confirm their account information through an 800 number or by logging in to a specific website. When an individual called in, a live agent would take the call and document 13 engagement characteristics (eTable 1 in Supplement 1). Website visits were also documented. Each mail piece came with a unique access code, and individuals who visited the website were identified if this access code was entered. On October 19, 2021, and November 15, 2021, emails containing the same information were sent to participants with email addresses on file (n = 223). Finally, between October 11, 2021, and November 24, 2021, 2 phone call attempts were made to each of the 644 participants. In these calls, a live agent again informed the individual that there was suspicious activity on the account and the reason for the call was to check whether the requested change to the account was legitimate. Responses to each outbound phone call were summarized into the same 13 engagement characteristics as the inbound calls.

### Engagement Groups

Based on participants’ responses to the 2 outbound call attempts as well as any inbound calls, we defined 3 engagement groups. Participants who neither answered the outbound phone call attempts nor called inbound to the 800 number were classified as no engagement. Participants who answered the phone or called in but were skeptical about the legitimacy of the outreach and did not give away personal information were classified as engagement. We defined skepticism as refusing to cooperate, questioning the caller’s intention, asking what USRPTF is, and refusing to allow call recording or give information. Third, participants who answered the phone or called in without skepticism; or confirmed that they did not change their account, names, or addresses; or provided the last 4 digits of their Social Security number were classified as conversion.

We focused our analyses on the data collected during the phone calls for 2 reasons. First, according to the Federal Trade Commission, phone calls are the most common and most effective method used by fraudsters for targeting older adults. Second, although we also conducted outreach via email, we found that a majority of the participants in this study did not have valid emails on file, and internet access was limited for many.

### Functional, Behavioral, and Psychosocial Measures

Participants underwent a comprehensive cognitive assessment and clinical evaluation for Alzheimer dementia. Financial decision-making and related behaviors, including financial literacy and scam awareness, were assessed (eMethods in Supplement 1). Depressive symptoms, loneliness, social networks, trust, and psychological well-being were also measured.^13,14,15,16,17^

### Statistical Analysis

Characteristics of the study participants were summarized using standard statistics. Group differences in demographics and functional and behavioral measures were assessed using analysis of variance, nonparametric Kruskal-Wallis test, χ^2^ test, or Fisher exact test as appropriate. Results showing an overall group difference were followed by post hoc pairwise comparisons. Statistical analyses were conducted using the SAS/STAT software, version 15.2 (SAS Institute) on a Red Hat Enterprise Linux server. Unless otherwise noted, statistical significance was determined at an α level of .05 for 2-sided tests.

## Results

### Characteristics of Study Participants

Of the 644 older adults included in this study, the mean (SD) age was 85.6 (7.5) years, 501 (77.8%) were female, and 595 (92.4%) were White. Participants received a mean of 16 years of education, and the median annual household income was between $50 000 and $75 000. Participants on average correctly answered 75% of the financial literacy questions and 4 of the 6 financial decision-making questions. On a scale of 1 to 7, with higher scores indicating lower awareness, the mean (SD) scam awareness score was 2.2 (0.8). Other characteristics are summarized in Table 1.

**Table 1.** Characteristics of the Study Participants (n = 644)

Characteristic	Mean (SD) [range]
Age, y	85.6 (7.5) [64.9 to 104.9]
Sex, No. (%)	
Female	501 (77.8)
Male	143 (22.2)
Education, y	16.0 (3.1) [8 to 30]
Race, No. (%)	
Black or African American	36 (5.6)
White	595 (92.4)
Other^a^	13 (2.0)
Income, median (IQR) [range]^b^	9 (7 to 10) [1 to 10]
Dementia, No. (%)	82 (12.7)
Cognition	−0.007 (0.90) [−3.9 to 1.9]
Depressive symptoms, median (IQR) [range]	1 (0 to 2) [0 to 9]
Loneliness	2.3 (0.7) [1 to 4.8]
Trust	24.2 (3.6) [7 to 32]
Social networks, median (IQR) [range]	4 (2 to 7) [0 to 51]
Psychological well-being	5.6 (0.6) [3.2 to 6.9]
Financial literacy	75.2 (18.4) [8.7 to 100]
Financial decision-making, median (IQR) [range]	4 (3 to 5) [0 to 6]
Scam awareness	2.2 (0.8) [1.0 to 5.2]
Temporal discounting (small stake), median (IQR) [range]	0.01 (0.005 to 0.02) [0.003 to 0.08]
Temporal discounting (large stake), median (IQR) [range]	0.35 (0.07 to 0.77) [0.07 to 2.73]
Risk aversion, median (IQR) [range]	0.08 (0.05 to 0.44) [0.04 to 0.91]
Financial fragility, No. (%)	27 (4.9)
Self-reported fraud victimization, No. (%)	56 (8.8)

^a^ The race category of “Other” includes American Indian or Alaska Native, Asian, Native Hawaiian or Other Pacific Islander, other, and unknown.

^b^ Income: 1: $0 to $4999, 2: $5000 to $9999, 3: $10 000 to $14 999, 4: $15 000 to $19 999, 5: $20 000 to $24 999, 6: $25 000 to $29 999, 7: $30 000 to $34 999, 8: $35 000 to $49 999, 9: $50 000 to $74 999, 10: $75 000 and over.

### Participants’ Responses to the Outreach

The mail pieces were sent to the 644 participants, and we could not confirm delivery to 3 participants because their mailers were not scanned on delivery. We emailed 223 participants with valid email addresses on file. Of those, 80 (35.9%) did not open the email, 143 (64.1%) opened the email, and 8 participants clicked the website link. A website was created for the experiment, and the website address was provided in the mailer and the email. Throughout the experiment, we recorded a total of 246 website visits. Unfortunately, since participants could only be identified if they also entered their unique access ID provided in the mailer and email, we were only able to identify 20 participants. Separately, a live agent called all 644 participants using the registered telephone numbers. During the first attempt, 125 participants answered the phone call, and an additional 61 participants answered the call during the second attempt. Seventeen participants called using the 800 number listed on the mailer and email. Together, 203 of 644 (31.5%) answered the phone calls or called in.

### Participants’ Engagement in the Experiment

A 3-level engagement measure was obtained based on the phone call records. Participants who neither answered the phone nor called in were defined as the no engagement group (n = 441, 68.5%). Participants who answered or called in but raised skepticism about the legitimacy of the outreach were defined as the engagement group (n = 97, 15.1%). Finally, participants who answered or called in without skepticism or provided personal information were defined as the conversion group (n = 106, 16.4%). Notably, 12 of the 17 participants (71%) who called in converted. In comparison, about 50% of the participants who answered the calls (94 of 186) converted. These data suggested that older adults who called in were more vulnerable to scams.

### Cognitive, Behavioral, and Psychosocial Vulnerabilities

We examined cognitive, behavioral, and psychosocial vulnerabilities that may place older adults at risk of victimization by comparing key characteristics across the 3 engagement groups. A few patterns emerged (Figure). First, participants in the engagement group had the highest cognition. In the post hoc pairwise comparisons adjusted for multiple testing, the mean cognitive score for the participants in the engagement group was about 0.24 (95% CI, 0.01-0.48) standard units higher than the no engagement group. The engagement group also had the lowest proportion of people with dementia in comparison to the other 2 groups (Table 2). Second, there were group differences in financial literacy. The engagement group scored highest in financial literacy. The mean financial literacy score for participants in the engagement group was 5.3 percentage points (95% CI, 0.5-10.1) higher than the no engagement group, and 7.2 percentage points (95% CI, 1.2-13.3) higher than the conversion group. Third, we also observed group differences in scam awareness. The result was primarily accounted for by the difference between the no engagement and conversion groups, and separately between the engagement and conversion groups. On average, participants in the conversion group scored lowest in scam awareness compared with the no engagement group (mean difference, 0.38; 95% CI, 0.18-0.59) or the engagement group (mean difference, 0.31; 95% CI, 0.03-0.58).

**Figure.**
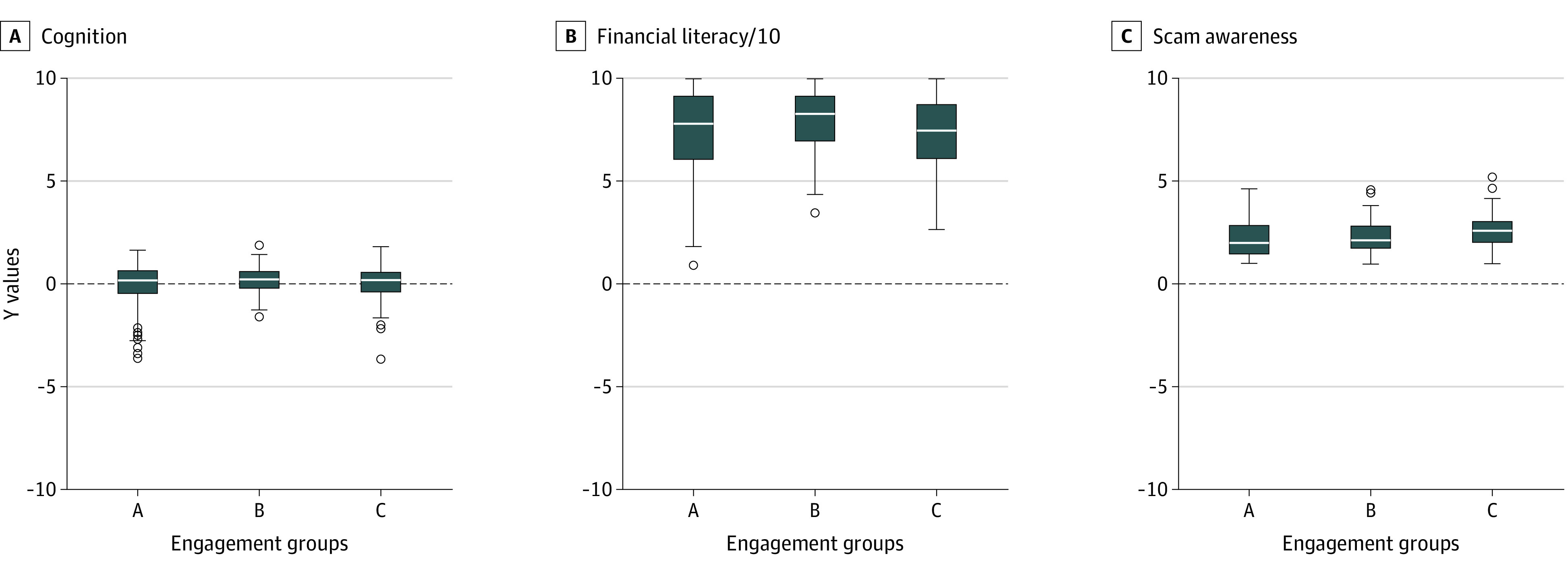
Factors Associated With Susceptibility to Government Impersonation Scams The figure illustrates the levels of cognition (A) (higher scores indicating higher cognition), financial literacy (B) (scaled by dividing original literacy scores by 10, with higher scores indicating higher financial literacy), and scam awareness (C) (higher scores indicating lower awareness) by engagement group (A: no engagement, B: engagement, C: conversion). Each panel is a boxplot, with the height of each box representing the IQR; line segment inside the box representing the median; whiskers representing the data range between Q1 − 1.5 × IQR and Q3 + 1.5 × IQR; and open circles representing outlier data points.

**Table 2.** Characteristics of the Study Participants by Engagement Groups

Characteristic	Mean (SD)			*P* value
	No engagement	Engagement	Conversion	
No.	441	97	106	NA
Age, y	85.5 (7.4)	85.8 (7.3)	86.2 (8.1)	.65^a^
Sex, No. (%)				
Female	346 (78.5)	70 (72.2)	85 (80.2)	.33^b^
Male	95 (21.5)	27 (27.8)	21 (19.8)	
Education, y	16.0 (3.1)	15.7 (2.8)	16.4 (3.2)	.27^a^
Race, No. (%)				
Black	23 (5.2)	4 (4.1)	9 (8.5)	.33^c^
White	408 (92.5)	93 (95.9)	94 (88.7)	
Other^d^	10 (2.3)	0	3 (2.8)	
Income, median (IQR)	8 (7 to 10)	9 (7 to 10)	9 (7 to 10)	.57^e^
Dementia, No. (%)	63 (14.3)	4 (4.1)	15 (14.2)	.02^b^
Cognition	−0.05 (1.0)	0.19 (0.6)	0.008 (0.8)	.05^a^
Depressive symptoms, median (IQR)	1 (0 to 2)	1 (0 to 2)	1 (0 to 2)	.97^e^
Loneliness	2.3 (0.7)	2.2 (0.6)	2.3 (0.6)	.90^a^
Trust	24.2 (3.6)	23.9 (3.7)	24.6 (3.7)	.36^a^
Social network, median (IQR)	5 (2 to 8)	5 (2 to 7)	4 (2 to 7)	.67^e^
Psychological well-being	5.6 (0.6)	5.7 (0.6)	5.6 (0.6)	.37^a^
Financial literacy	74.7 (19.1)	80.0 (14.7)	72.8 (18.1)	.01^a^
Financial decision-making, median (IQR)	4 (3 to 5)	4 (3 to 5)	4 (2 to 4)	.14^e^
Scam awareness	2.17 (0.8)	2.25 (0.8)	2.55 (0.8)	<.001^a^
Temporal discounting (small stake), median (IQR)	0.01 (0.005 to 0.02)	0.005 (0.003 to 0.02)	0.01 (0.005 to 0.02)	.08^e^
Temporal discounting (large stake), median (IQR)	0.35 (0.16 to 0.77)	0.17 (0.07 to 0.77)	0.35 (0.16 to 0.77)	.11^e^
Risk aversion, median (IQR)	0.08 (0.05 to 0.47)	0.08 (0.05 to 0.33)	0.08 (0.05 to 0.55)	.26^e^
Financial fragility, No. (%)	19 (5.1)	2 (2.3)	6 (6.1)	.42^c^
Self-reported fraud victimization, No. (%)	37 (8.5)	12 (12.6)	7 (6.7)	.30^b^

Abbreviation: NA, not applicable.

^a^ Analysis of variance.

^b^ χ^2^ test.

^c^ Fisher exact test.

^d^ The race category of “Other” includes American Indian or Alaska Native, Asian, Native Hawaiian or Other Pacific Islander, other, and unknown.

^e^ Kruskal-Wallis test.

In a sensitivity analysis, we excluded individuals with dementia (n = 82). Participants in the conversion group still scored lowest in scam awareness (eTable 2 in Supplement 1). The mean financial literacy and, separately, cognitive scores remained the highest in the engagement group, but the differences did not reach statistical significance.

## Discussion

We conducted a behavioral experiment designed to assess susceptibility to financial fraud and scams among community-dwelling older adults. Our results revealed that a sizable number of older adults (16.4%) engaged without skepticism, and of those, nearly three-quarters provided personal information. Some (15.1%) engaged but were potentially alert to the fraudulent nature of the outreach and did not confirm or share personal information. A majority (68.5%) did not engage in any capacity. Further examinations of functional, behavioral, and psychosocial characteristics revealed that cognition, financial literacy, and scam awareness are important factors associated with vulnerability.

Our research experiment was framed specifically around a fictitious government agency inquiring about a possible retirement benefit account breach. Government impersonation is among the most common scams that target older adults. According to the elder fraud report published by the Federal Bureau of Investigation, in 2021, over 3000 individuals 60 years and older lost money to government impersonation fraud. Between 2015 and 2020, of the total 8402 complaints reported to the fraud hotline of US Senate Special Committee on Aging, 3383 (40.3%) were government impersonation scams.^18^

The results from our study provide novel data on fraud victimization in the context of a government impersonation scam against older adults. Alarmingly, approximately 16% engaged without skepticism in conversations with an agent impersonating a government representative. Even more concerning, 12% of the participants in the experiment willingly shared personal information, and close to 5% provided the last 4 digits of their Social Security number. If extrapolated to a population level, these numbers are astounding and suggest that a very large number of older adults are at risk of victimization, far exceeding findings previously observed in survey data. These estimates likely are on the low side given that we used a fictitious government agency name. Fraudsters create more compelling scams by impersonating real government agencies and organizations.

To date, studies on financial fraud and scam victimization among older adults have relied almost exclusively on self-reported data. While self-reported data are valuable, dependence on subjective perceptions and memories make it vulnerable to biases. Older adults may be unaware that they were victimized, may have forgotten the incident, or may be unwilling to acknowledge victimization due to shame or concerns that it adversely reflects on their intelligence, perceived cognition, or decision-making and, consequently, could compromise their independence. In a recent review on the prevalence of financial fraud and scam victimization^1^ including a meta-analysis of 12 studies, all of which used data obtained via in-person or phone interviews, all 12 studies were rated as having moderate to high risk of bias. By contrast, the current study exposed participants to deceptive materials and then assessed their levels of engagement. As a result, our study design circumvents recall, social desirability, and other response biases that typically arise during survey or interview approaches. Thus, these data provide more objective and accurate results regarding older adults’ susceptibility to fraud and scams.

Further, our study suggests that older adults in different engagement groups had varying levels of cognition, financial literacy, and scam awareness. These results are largely consistent with the very limited prior literature, including studies from the current cohort.^19,20^ Individuals in the engagement group scored the highest in financial literacy compared with the no engagement or conversion group. A similar result was also observed in cognition. Separately, older adults in the conversion group were least aware of scams, and there was no statistical difference between the no engagement and engagement groups. Together, the data suggest that older adults with higher levels of financial literacy or cognition are potentially more capable of detecting fraudulent outreaches; on the other hand, it is quite clear that low awareness of scams renders older adults highly susceptible to fraud and scams.

The result that older adults in the engagement group had higher financial literacy and cognition than the no engagement group is intriguing, considering that nonengagement is far and away the best strategy of preventing fraud victimization. One possibility is that a person needs to be cognitively functional enough to respond to any solicitation, whether it is legitimate or fraudulent, and individuals with severe cognitive impairment may lack the capability to engage. The result was inconclusive if we exclude individuals with dementia. Further investigations are warranted.

To our knowledge, this is the first study that objectively examined financial fraud and scams in older adults by means of a behavioral experiment. The study design minimizes the biases inherent in survey or interview data and therefore provides extremely novel and more accurate data on scam susceptibility. Further, participants came from an existing cohort study of aging that systematically collects a wide range of functional, behavioral, and psychosocial information about community-living older adults, and this allowed for a careful investigation of the factors that may render older adults vulnerable to fraud and scams. Importantly, as most survey- or interview-based fraud studies do not recruit the oldest old or individuals with cognitive impairment, our study also informs on a group of individuals who potentially are most vulnerable to financial fraud and scams.

### Limitations

MAP participants are predominantly White with high education. Consequently, findings reported in this work may not generalize to the general aging population. It is noteworthy, however, that the relatively high percentage of people who engaged in this study is likely an underestimation considering this highly educated and restricted sample. Further, the agent in the study did not apply the same high-pressure persuasion tactics that most fraudsters would use when engaging older adults, and hence the rate of conversion likely would have been much higher in the real world.

## Conclusions

The findings of this cross-sectional study provide powerful evidence that many more older adults than currently recognized, including many without cognitive impairment, actively engage with potentially fraudulent pitches and are at risk of victimization and the deleterious health and financial consequences that result.

## Notes

Supplement 1.
eMethods.
**eFigure 1.** Sample Size Flowchart **eFigure 2.** Template of the Mailer and Email Used in the Behavioral Experiment **eTable 1.** Engagement Characteristics Documented During the Phone Calls **eTable 2.** Characteristics of the Study Participants (Nondemented)

Supplement 2.
Data Sharing Statement
