# Supplementary material for: Vulnerability of Older Adults to Government Impersonation Scams
Source: JAMA Netw Open. 2023 Sep 22;6(9):e2335319. doi: 10.1001/jamanetworkopen.2023.35319 (PMC10517371; doi:10.1001/jamanetworkopen.2023.35319)
Supplement: Supplement 1. — eMethods. eFigure 1. Sample Size Flowchart eFigure 2. Template of the Mailer and Email Used in the Behavioral Experiment eTable 1. Engagement Characteristics Documented During the Phone Calls eTable 2. Characteristics of the Study Participants (Nondemented) [file jamanetwopen-e2335319-s001.pdf]

Supplemental Online Content

Yu L, Mottola G, Kieffer CN, et al. Vulnerability of older adults to government impersonation scams. *JAMA Netw Open.* 2023;6(9):e2335319.
doi:10.1001/jamanetworkopen.2023.35319

eMethods.

eFigure 1. Sample Size Flowchart

eFigure 2. Template of the Mailer and Email Used in the Behavioral Experiment

eTable 1. Engagement Characteristics Documented During the Phone Calls

eTable 2. Characteristics of the Study Participants (Nondemented)

This supplemental material has been provided by the authors to give readers additional information about their work.

© 2023 Yu L et al. *JAMA Network Open.*

eMethods.

Financial Decision Making and Related Behaviors

Financial literacy, financial decision making, scam awareness, self-reported fraud victimization, financial fragility, temporal discounting and risk aversion were assessed annually, as previously described[1-8].

Briefly, financial literacy was assessed using a 23-item instrument that covered questions on financial knowledge, numeracy and investments. A financial literacy score was calculated as the percentage of correct answers to these 23 items (Range: 0-100). Financial decision making was assessed using a 6-item module that mimicked real world financial decisions. Participants were shown tables with financial information about mutual funds and asked questions with varying levels of difficulty, which range from simply exacting the information presented in the table to integrating information in order to pick a mutual fund that met multiple criteria. Financial decision making was scored as the total number of correct answers (Range: 0-6).

Scam awareness was measured via a 5-item instrument. Participants rated agreement on each item using a 7-point scale. Questions (e.g., answering a phone call from a stranger or ending a call from a telemarketer) were selected based on prior findings from the FINRA Foundation in collaboration with the American Association of Retired Persons, and aimed to capture behaviors commonly associated with fraud and scams victimization. Item-specific ratings were averaged to a summary score, with higher scores indicating low scam awareness. Separately, participants were asked whether they had been a victim of financial fraud within the past year. Financial fragility was assessed by asking the participants if they could come up with $2,000 within a month to pay for an unexpected expense.

© 2023 Yu L et al. *JAMA Network Open.*

Temporal discounting refers to the tendency to prefer a fixed reward now over a larger reward at a later time. Two temporal discounting measures, one involving a small dollar amount and one involving a large dollar amount, were derived using an established hyperbolic function[9] that estimates the odds of choosing the delayed reward at varying values over an immediate reward. Risk aversion refers to the tendency to prefer a certain reward over an unknown but possibly larger reward. The measure was estimated using questions that involve choices between taking a certain reward versus gambling for a possibly larger reward by flipping a coin. For both measures, higher values indicate more discounting or more risk averse.

Age, sex, education, and race

Age was calculated using date of birth and a date in the middle of the behavioral experiment (i.e., 11/01/2021). Sex, number of years of regular school, and race were reported by the participants at the baseline interview of the parent study. The race classification reflects the revised National Institutes of Health (NIH) standards, which include 7 categories of White, Black or African American, American Indian or Alaska Native, Native Hawaiian or Other Pacific Islander, Asian, Other, and Unknown.

eReferences

1. Boyle PA, Yu L, Schneider JA, Wilson RS, Bennett DA. Scam Awareness Related to Incident Alzheimer Dementia and Mild Cognitive Impairment: A Prospective Cohort Study. *Annals of internal medicine.* 2019;170(10):702-709.
2. Lamar M, Yu L, Leurgans S, et al. Self-reported fraud victimization and objectively measured blood pressure: Sex differences in post-fraud cardiovascular health. *Journal of the American Geriatrics Society.* 2022;70(11):3185-3194.
3. Yu L, Mottola G, Barnes LL, et al. Financial fragility and scam susceptibility in community dwelling older adults. *J Elder Abuse Negl.* 2022;34(2):93-108.
4. Glover CM, Yu L, Stewart CC, Wilson RS, Bennett DA, Boyle PA. The Association of Late Life Cognitive Activity with Healthcare and Financial Decision-Making in Community-Dwelling, Nondemented Older Adults. *The American journal of geriatric psychiatry : official journal of the American Association for Geriatric Psychiatry.* 2021;29(2):117-125.
5. Wilson RS, Yu L, Schneider JA, Bennett DA, Boyle PA. Risk Aversion and Alzheimer Disease in Old Age. *The American journal of geriatric psychiatry : official journal of the American Association for Geriatric Psychiatry.* 2019;27(8):851-861.
6. Stewart CC, Yu L, Wilson RS, Bennett DA, Boyle PA. Healthcare and Financial Decision Making and Incident Adverse Cognitive Outcomes among Older Adults. *Journal of the American Geriatrics Society.* 2019;67(8):1590-1595.
7. Han SD, Arfanakis K, Fleischman DA, Yu L, Bennett DA, Boyle PA. White matter correlates of temporal discounting in older adults. *Brain structure & function.* 2018;223(8):3653-3663.
8. Weissberger GH, Han SD, Yu L, Barnes LL, Bennett DA, Boyle PA. Financial and health literacy discrepancies with cognition in older adults. *Neuropsychology.* 2019;33(7):975-985.
9. Laibson D. Golden eggs and hyperbolic discounting. *The Quarterly Journal of Economics.* 1997;112(2):443-478.

© 2023 Yu L et al. *JAMA Network Open.*

eFigure 1. Sample Size Flowchart

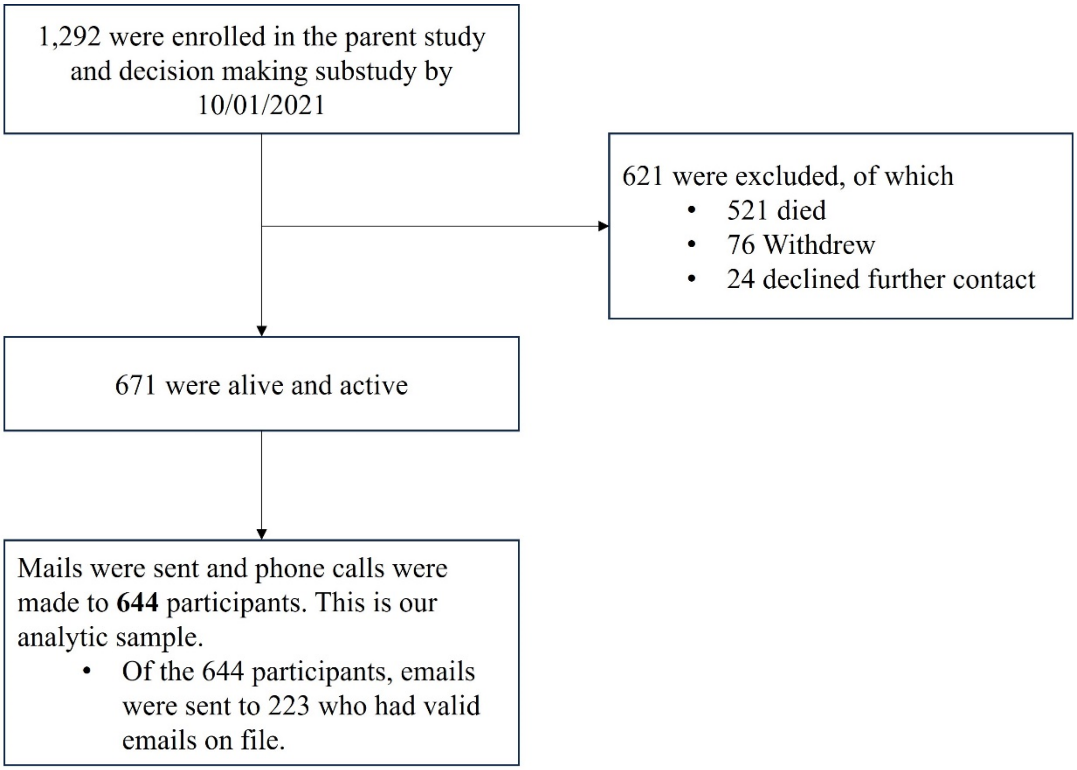

© 2023 Yu L et al. *JAMA Network Open.*

eFigure 2. Template of the Mailer and Email Used in the Behavioral Experiment

U.S. RETIREMENT
PROTECTION TASK FORCE

OFFICIAL FINAL NOTICE

Attention,

First/Last Name

The U.S. RETIREMENT PROTECTION TASK FORCE is contacting you **FOR THE FINAL TIME** about a possible account breach in one or more of your retirement-related accounts. Our records show that we attempted to contact you once by mail, twice by email and twice by telephone in October/November of last year. **SINCE THAT TIME, WE'VE SEEN CHANGES TO YOUR ACCOUNT INFORMATION THAT WERE MADE FROM AN OUT OF STATE COMPUTER.**

We need to confirm whether those changes were authorized. If they were not, we need to confirm some details in order to secure your file in order to protect your retirement services such as Medicare and Social Security.

The easiest way to confirm your account information is to call the phone number listed below and speak to one of our representatives. This only takes 5 minutes and will ensure that your retirement accounts are secure.

Phone: 1-877-312-2804
Hours: Monday – Friday, 8:30 am – 5:00 pm (Eastern Daylight Time)

If you prefer to confirm your account online, use our secure, encrypted Account Confirmation page by visiting the website listed below. If verifying online, you will need to type in the 8-digit code listed below as a security measure.

Website: WWW.USRPTF.COM

CODE: 69958519

ABOUT THE U.S. RETIREMENT PROTECTION TASK FORCE

As part of a new response protocol implemented in the wake of a series of retirement-related account breaches in 2018 and 2019, the U.S. Retirement Protection Task Force is responsible for ascertaining and maintaining regularly updated benefit program account files, including occasional requests for confirmation from beneficiaries, as well as the implementation of the latest security measures as they become available. During the COVID-19 crisis, retirement fraud spiked as various organizations seeking to defraud elder citizens used the crisis as an opportunity to pray on anxieties arising from the pandemic. These events have made our efforts even more important and have accelerated the immediacy of our attempts to collect the information required to secure files for the rightful parties.

© 2023 Yu L et al. *JAMA Network Open.*

eTable 1. Engagement Characteristics Documented During the Phone Calls

Characteristics	N (%)
#1: Live Answer	203 (32.0%)
#2: Left VM	266 (41.3%)
#3: Refused call recording	5 (0.8%)
#4: Hung up; refused to cooperate	106 (16.4%)
#5: Confirmed they didn't change account email	73 (11.3%)
#6: Confirmed full name	68 (10.6%)
#7: Confirmed address	66 (10.3%)
#8: User provided last 4 digits of SSN	31 (4.8%)
#9: Questioned if caller was trying to defraud them	27 (4.2%)
#10: Asked what USRPTF is	35 (5.4%)
#11: Seemed concerned that their account had already been compromised	44 (6.8%)
#12: Didn't want to give info over the phone	25 (3.9%)
#13: Said they would confirm online	2 (0.3%)

Note. An agent from Heart and Mind Strategies who handled the call would checkmark each of the fields based on the conversation.

© 2023 Yu L et al. *JAMA Network Open.*

eTable 2. Characteristics of the Study Participants (Nondemented)

	No Engagement	Engagement	Conversion	*p*
N	378	93	91	
Age	84.8 (7.3)	85.5 (7.3)	85.3 (7.8)	0.64§
Sex				
Female sex	299 (79.1%)	67 (72.0%)	72 (79.1%)	0.32◊
Male sex	79 (20.9%)	26 (28.0%)	19 (20.9%)	
Education	15.9 (3.1)	15.8 (2.8)	16.4 (3.3)	0.38§
Race				
Black or African Americans	15 (4.0%)	4 (4.3%)	7 (7.7%)	0.29▲
White	353 (93.4%)	89 (95.7%)	82 (90.1%)	
Other	10 (2.6%)	0 (0%)	2 (2.2%)	
Income	9 (7-10)	9 (7-10)	9 (7-10)	0.69†
Cognition	0.22 (0.6)	0.24 (0.6)	0.21 (0.6)	0.94§
Depressive symptoms	1 (0-2)	1 (0-2)	1 (0-2)	0.97†
Loneliness	2.2 (0.7)	2.2 (0.6)	2.2 (0.5)	0.83§
Trust	24.3 (3.7)	23.9 (3.7)	24.5 (3.7)	0.47§
Social networks	5 (2-8)	5 (2-7)	4 (2-7)	0.69†
Psychological wellbeing	5.6 (0.6)	5.7 (0.6)	5.6 (0.6)	0.57§
Financial literacy	77.8 (17.3)	80.7 (14.6)	76.5 (15.4)	0.19§
Financial decision making	4 (3-5)	4 (3-5)	4 (3-4)	0.19†
Scam awareness	2.05 (0.8)	2.21 (0.8)	2.46 (0.8)	<.001§
Temporal discounting (small stake)	0.01 (0.005-0.02)	0.005 (0.003-0.01)	0.01 (0.003-0.02)	0.16†
Temporal discounting (large stake)	0.35 (0.07-0.77)	0.16 (0.07-0.77)	0.35 (0.16-0.77)	0.14†
Risk aversion	0.08 (0.05-0.43)	0.08 (0.05-0.29)	0.08 (0.05-0.43)	0.31†
Financial fragility	17 (5.1%)	2 (2.4%)	4 (4.8%)	0.66▲
Self-reported fraud victimization	35 (9.3%)	12 (13.2%)	7 (7.7%)	0.42◊

© 2023 Yu L et al. *JAMA Network Open.*

Income: 1: $0 - $4,999 2:$5,000 - $9,999, 3: $10,000 - $14,999, 4: $15,000 - $19,999, 5:$20,000 - $24,999, 6: $25,000 - $29,999, 7:$30,000 - $34,999, 8: $35,000 - $49,999, 9: $50,000 - $74,999, 10: $75,000 and over.

Note. [§]ANOVA; [◊]Chi squared test; [†] Kruskal-Wallis test; [▲]Fisher exact test.

© 2023 Yu L et al. *JAMA Network Open.*
