# Supplementary material for: Vulnerability of Older Adults to Government Impersonation Scams
Source: JAMA Netw Open. 2023 Sep 22;6(9):e2335319. doi: 10.1001/jamanetworkopen.2023.35319 (PMC10517371; doi:10.1001/jamanetworkopen.2023.35319)
Supplement: Supplement 2. — Data Sharing Statement [file jamanetwopen-e2335319-s002.pdf]

Data Sharing Statement

Yu. Vulnerability of Older Adults to Government Impersonation Scams. *JAMA Netw Open*. Published September 22, 2023. doi:10.1001/jamanetworkopen.2023.35319

Data
Data available: Yes
Data types: Deidentified participant data
How to access data: Data used in this study can be requested through the RADC Research Resource Sharing Hub at https://www.radc.rush.edu.
When available: With publication

Supporting Documents
Document types: None

Additional Information
Who can access the data: researchers whose proposed use of the data has been approved
Types of analyses: for research purposes only
Mechanisms of data availability: The data request will be reviewed and voted on by a RADC committee, and if approved, data will be shared with the requestor after the establishment of a data use agreement between their institution and RADC.
